# Dietary patterns before and during pregnancy and small for gestational age in Japan: a prospective birth cohort study

**DOI:** 10.1186/s12937-022-00808-7

**Published:** 2022-09-16

**Authors:** Takahiro Yamashita, Taku Obara, Yudai Yonezawa, Ippei Takahashi, Mami Ishikuro, Keiko Murakami, Fumihiko Ueno, Aoi Noda, Tomomi Onuma, Noriyuki Iwama, Hirotaka Hamada, Junichi Sugawara, Shigenori Suzuki, Hiroyuki Suganuma, Masatoshi Saito, Nobuo Yaegashi, Shinichi Kuriyama

**Affiliations:** 1 Division of Molecular Epidemiology, Department of Preventive Medicine and Epidemiology, Tohoku Medical Megabank Organization, Tohoku University, 2-1 Seiryo-machi, Aoba-ku, Sendai, 980-8573 Japan; 2 Innovation Division, KAGOME CO., LTD, Nasushiobara, Japan; 3 Division of Molecular Epidemiology, Environment and Genome Research Center, Graduate School of Medicine, Tohoku University, Sendai, Japan; 4 Department of Pharmaceutical Sciences, Tohoku University Hospital, Sendai, Japan; 5 Department of Gynecology and Obstetrics, Graduate School of Medicine, Tohoku University, Sendai, Japan; 6 Department of Community Medical Supports, Tohoku Medical Megabank Organization, Tohoku University, Sendai, Japan; 7 Tohoku Medical Megabank Organization, Tohoku University, Sendai, Japan; 8 Division of Disaster Public Health, International Research Institute of Disaster Science, Tohoku University, Sendai, Japan

**Keywords:** Birth weight, Dietary patterns, Partial least squares, Maternal nutrition, Pregnancy, Principal component analysis, Prospective birth cohort studies, Reduced rank regression, Small for gestational age

## Abstract

**Background:**

Although small for gestational age (SGA) is a serious problem worldwide, the association of dietary patterns before and during pregnancy with SGA risk is unclear. We evaluated this association among Japanese pregnant women using three methods: reduced rank regression (RRR) and partial least squares (PLS), methods for extracting dietary patterns that can explain the variation of response variables, and principal component analysis (PCA), a method for extracting dietary patterns of the population.

**Methods:**

Between July 2013 and March 2017, 22,493 pregnant women were recruited to the Tohoku Medical Megabank Project Birth and Three-Generation Cohort Study, a population-based prospective birth cohort study in Japan. Information on dietary intake was obtained using food frequency questionnaires, and dietary patterns were extracted using RRR, PLS, and PCA. Information on birth weight was obtained from obstetric records, and the birth weight SD score and SGA were defined by the method of the Japan Pediatric Society. The associations of dietary patterns with birth weight SD score and SGA risk were investigated using multiple linear regression and multiple logistic regression, respectively.

**Results:**

A total of 17,728 mother-child pairs were included. The birth weight SD score was 0.15 ± 0.96, and the prevalence of SGA was 6.3%. The dietary patterns extracted by RRR and PLS were similar and characterized by a high intake of cereals and fruits and a low intake of alcoholic and non-alcoholic beverages in both pre- to early pregnancy and from early to mid-pregnancy. Higher adoption of the RRR and PLS patterns in both periods was associated with an increased birth weight SD score and lower risk of SGA. In contrast, the PCA1 pattern was not associated with birth weight SD score or SGA risk in either period. Although the PCA2 pattern was associated with increased birth weight SD score from early to mid-pregnancy, no other associations with birth weight SD score or SGA risk were observed.

**Conclusions:**

The dietary pattern with a high intake of cereals and fruits and a low intake of alcoholic and non-alcoholic beverages before and during pregnancy was associated with a decreased SGA risk in Japan.

**Supplementary Information:**

The online version contains supplementary material available at 10.1186/s12937-022-00808-7.

## Background

Small for gestational age (SGA) is defined as a birth weight below the 10th percentile of the reference birth weight by sex and weeks of gestation in a region [1]. SGA has long-term effects on individual health status, including the risk of neonatal mortality as well as the risk of stunting in childhood and metabolic syndrome in adulthood, and continues to be an important global public health issue [2–4]. In Japan, particularly, it has been reported that undereating owing to the desire to stay slim among young women has become a problem, which is spurring the downward trend in the birth weights of their infants [5].

Maternal nutritional status is considered one of the most important factors in healthy fetal development [6, 7]. However, although many epidemiological studies have examined the association between food and nutrient intake during pregnancy, such as fruits, vegetables, fish, dairy products, carbohydrates, fats, and vitamins with fetal development, inconsistent results have been reported [8–10]. One possible reason for this is that people usually consume a wide variety of foods containing various nutrients in daily life. In other words, studies of single foods and nutrients may not consider the synergistic and counteracting effects of foods and nutrients on the body. In light of these issues, a method that captures food intake as a pattern is considered useful for studying the association between diet and diseases [11].

One of the major methods for extracting dietary patterns is principal component analysis (PCA). PCA derives a linear function of food intake that best explains the variation in food intake and can reflect people’s actual dietary patterns [12]. However, they do not consider the association with diseases, and thus may not be associated with disease risk [11]. Reduced rank regression (RRR) and partial least squares (PLS) have been proposed as alternatives to PCA [13, 14]. RRR is a method for extracting dietary patterns that best explain variation in response variables (e.g., asymptomatic or clinical endpoints and disease-related nutrients) [12]. The dietary patterns extracted by RRR are more relevant to disease risk than PCA. However, they do not consider the variation in food intake as those of PCA, thus they do not necessarily reflect people’s actual dietary patterns [11]. Therefore, RRR and PCA complement each other, and useful insights can be gained by comparing the results [12]. PLS is a compromise method between RRR and PCA, which is a method for extracting dietary patterns that best explain the variation in food intake and response variables [13].

Several reports have investigated the association between dietary patterns and SGA in pregnant women using PCA. Knudsen et al. reported in a prospective study in Denmark that the “health-conscious pattern” characterized by a high intake of vegetables, fruits, fish, and poultry was associated with a lower risk of SGA [15]. Thompson et al. reported in a case-control study in New Zealand that the “traditional pattern” characterized by high intake of apples/pears, citrus fruits, kiwi/feijoa, bananas, green vegetables, root vegetables, beans/corn, dairy products/yogurt, and water was associated with a lower risk of SGA [16]. In contrast, a prospective study in Singapore by Chia et al. and a retrospective cross-sectional study in Australia by Grieger et al. reported that there were no dietary patterns that were significantly associated with SGA risk [17, 18]. Thus, there are no consistent results regarding the association of dietary patterns extracted by PCA with SGA risk among pregnant women. According to the PCA, the reason for this may be that people’s actual dietary patterns during pregnancy may have little or no association with SGA.

However, to date, there has been one report that used RRR to evaluate the association between dietary patterns and SGA in pregnant women. A prospective study in China by Liu et al. reported that a dietary pattern characterized by a high intake of legumes, soy products, vegetables, and animal products and a low intake of wheat and oil was associated with a lower risk of SGA [19]. However, they did not assess the association of dietary patterns with SGA using the PCA and did not compare the results with those obtained using RRR thus, it is not clear to what extent the dietary patterns extracted by RRR were similar to the actual dietary patterns of people. Therefore, in this study, we used RRR and PLS to determine which dietary patterns are associated with SGA risk in Japanese pregnant women and compared them with PCA to determine how similar they are to people’s current actual diets.

## Methods

### Study design and participants

**Fig. 1:**
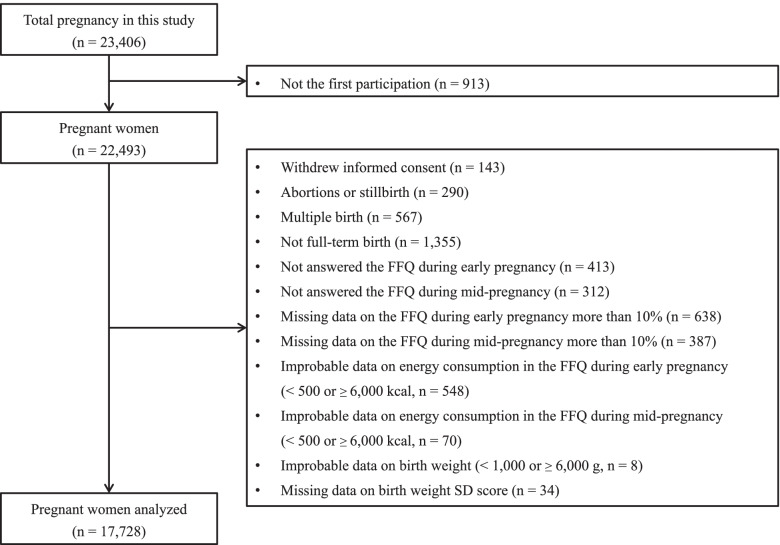
Selection flow chart of participants in this study. The flow chart describes the exclusion criteria and the total number of participants, excluded participants, and eligible participants. FFQ: food frequency questionnaire

This study used data from the Tohoku Medical Megabank Project Birth and Three-Generation Cohort Study (TMM BirThree Cohort Study), which is a population-based prospective birth cohort study conducted by the Tohoku Medical Megabank Organization (ToMMo). This study aimed to facilitate the resolution of medical issues following the Great East Japan Earthquake of March 11, 2011, and the details of the study described previously [20, 21]. Briefly, 22,493 pregnant women in early pregnancy were recruited between July 2013 and March 2017 from approximately 50 obstetric clinics and hospitals in both rural and urban areas of Miyagi prefecture, Japan. Written informed consent was obtained from all participants. The TMM BirThree Cohort Study was approved by the Ethics Committee of ToMMo (2013–1–103-1) and was conducted per the Declaration of Helsinki. A dataset fixed on January 31, 2019, was used for our analysis, and the selection of subjects is shown in the flowchart in Fig. 1.

### Birth weight Standard Deviation (SD) score and SGA

Birth weight, sex of the child, parity, and gestational age at birth were obtained from obstetric records at birth. The birth weight SD score was defined as the SD of the distribution of birth weight considering the sex of the child, parity, and gestational age at birth in accordance with the method of the Japan Pediatric Society and SGA was defined as a birth weight below the 10th percentile of Japanese infants [22].

### Dietary information

Details of dietary information are described in Supporting information 1. Briefly, to investigate dietary intake before and during pregnancy, we used two semi-quantitative food frequency questionnaires (FFQs). The daily intakes of 13 food groups and 28 nutrients from pre- to early pregnancy and from early to mid-pregnancy obtained by the FFQs were used in this study. The intake of each food group and nutrient was adjusted for energy intake using the residual method [23] and then standardized by subtracting the mean value and dividing by the standard deviation of each corresponding food group and nutrient intake.

### Dietary patterns

Details of dietary patterns are described in Supporting information 2. Briefly, dietary patterns from pre- to early pregnancy and from early to mid-pregnancy were extracted using RRR, PLS, and PCA using PROC PLS in SAS (version 9.4, SAS Institute Inc., Cary, North Carolina) with selecting the intake of 13 food groups as predictor variables and birth weight SD score as a response variable, which was a continuous variable associated with SGA risk. Factor loadings on food groups obtained by RRR, PLS, and PCA represent the degree of contribution of that food group to the dietary pattern [24]. Food groups with absolute factor loadings of 0.2 or higher were considered to characterize the dietary pattern [12]. The factor scores of each subject indicated the degree of adoption of the dietary pattern. They were divided into quartiles (Quartile 1: lowest adoption; Quartile 4: highest adoption) and used for analysis in association with birth weight SD score and SGA risk.

### Potential confounders

Details of potential confounders are described in Supporting information 3. Briefly, maternal age at delivery, pre-pregnancy body mass index (BMI), maternal weight gain during pregnancy, parity, educational qualification, annual household income, cigarette smoking status, alcohol drinking, and folic acid supplement consumption during early pregnancy were considered as potential confounders for the association between dietary patterns and birth outcomes based on previous reports [8, 25, 26].

### Statistical analysis

Participants’ background attributes are presented as mean ± SD for continuous variables and as frequency (percentage) for categorical variables. Comparisons of the background attributes stratified by dietary patterns were assessed by ANOVA and *P* for trend for continuous variables and by χ^2^ test or Cochran-Armitage test for categorical variables.

The association of each dietary pattern with birth weight SD score was assessed using multiple regression analysis. The association between each dietary pattern and SGA risk was assessed using logistic regression analysis. In model 1, we adjusted for maternal age, pre-pregnancy BMI, educational qualification, annual household income, smoking habits, drinking habits, and folic acid supplement consumption habits. We did not adjust for maternal weight gain during pregnancy in model 1 because we assumed that maternal weight gain might be an intermediate variable between dietary patterns and outcomes. However, in model 2, we adjusted for maternal weight gain to estimate the association between dietary patterns and the outcomes, independent of maternal weight gain. The missing values for the confounding variables were complemented using the multivariate completion method with chained equations, where the missing data were assumed to be random, using the mice package in R [27]. The number and percentage of missing data in the confounding variables are shown in Table 1. Five independent datasets with complementary missing values were created. Multivariate analysis was performed on each dataset and the estimates of each result were combined.

**Table 1:** Characteristics of participants (*n* = 17,728)

Variables	Mean or n	SD or %^a^
**Maternal characteristics**		
Maternal age (years), n and %		
< 25	1283	7.2
25–29	4493	25.3
30–34	6572	37.1
≧ 35	5380	30.3
Number of missing values	0	0
Pre-pregnancy body mass index (kg/m^2^), mean and SD	21.6	3.4
Number of missing values, n and %	228	1.3
Maternal gestational weight gain (kg), mean and SD	10.1	4.2
Number of missing values, n and %	4152	23.4
Gestational age (week), mean and SD	39.0	1.2
Number of missing values, n and %	0	0
Parity, n and %		
One or more	9173	51.7
Never	8555	48.3
Number of missing values	0	0
Educational qualification, n and %		
High school graduate or less	3676	20.7
College graduate	4417	24.9
University graduate or above	3313	18.7
Number of missing values	6322	35.7
Annual household income (Japanese yen/year), n and %		
< 4,000,000	6108	34.5
4,000,000–5,999,999	5525	31.2
≧ 6,000,000	5287	29.8
Number of missing values	808	4.6
Cigarette smoking, n and %		
Never	10,698	60.3
Quit before pregnancy	4075	23.0
Quit after noticing pregnancy	2487	14.0
Current	410	2.3
Number of missing values	58	0.3
Alcohol consumption, n and %		
Never	8016	45.2
Former	6118	34.5
Current	3547	20.0
Number of missing values	47	0.3
Folic acid supplement consumption during early pregnancy, n and %		
Yes	9990	56.4
No	7708	43.5
Number of missing values	30	0.2
**Child characteristics**		
Sex, n and %		
Boys	9159	51.7
Girls	8569	48.3
Number of missing values	0	0
Birth weight (g), mean and SD	3072	368
Number of missing values, n and %	0	0
Birth weight SD score, mean and SD	0.15	0.96
Number of missing values, n and %	0	0
Small for gestational age, n and %		
Yes	1116	6.3
No	16,612	93.7
Number of missing values	0	0

*SD* standard deviation

^a^Frequency percentages include the number of missing values

To assess the robustness of the extracted dietary patterns, we performed some sensitivity analyses. To evaluate the possibility of selection bias, RRR, PLS, and PCA were performed, and dietary patterns were reextracted with the excluded subjects who had available data on dietary intake and incidence of SGA (*n* = 18,667). RRR, PLS, and PCA were also performed in the included subjects stratified by maternal characteristics such as maternal age (< 30 and ≥ 30 years), annual household income (< 4,000,000, 4,000,000–5,999,999, and ≥ 6,000,000 Japanese yen per year), and educational qualification (high school graduate or less, college graduate, and university graduate or above). We also performed RRR, PLS, and PCA in subjects without gestational diabetes mellitus (GDM) to exclude possible confounding of the effects of GDM on SGA risk (*n* = 17,325).

SAS ver. 9.4 was used for extracting dietary patterns, and R ver. 4.0.1 was used for all other statistical analyses. Statistical significance was set at *P* <  0.05.

## Results

### Characteristics of participants

A total of 17,728 mother-child pairs were included in the analysis. Approximately two-thirds (67.4%) of mothers were over 30 years old, with a mean pre-pregnancy BMI of 21.6 ± 3.4 kg/m^2^, mean weight gain of 10.1 ± 4.2 kg during pregnancy, and mean gestational age of 39.0 ± 1.2 weeks (Table 1). Approximately half of the women were nulliparous at enrolment in the study (48.3%) and were taking folic acid supplements during early pregnancy (56.4%). Few mothers continued to smoke during early pregnancy (2.3%), while a large number of mothers continued to drink alcohol during early pregnancy (20.0%). The mean birth weight SD score was 0.15 ± 0.96, and the prevalence of SGA was 6.3% (*n* = 1116).

### Dietary patterns identified by RRR, PLS, and PCA

The factor loadings and explained variation of the dietary patterns extracted by RRR, PLS, and PCA from pre- to early pregnancy and from early to mid-pregnancy are shown in Table 2 and Supplementary Table 1, respectively. The RRR and PLS patterns were both characterized by a high intake of cereals and fruits and a low intake of alcoholic and non-alcoholic beverages from pre- to early pregnancy and from early to mid-pregnancy. Additionally, the PLS pattern from early to mid-pregnancy was characterized by a high intake of dairy products and mushrooms. The PCA1 pattern was characterized by a high intake of legumes, vegetables, fruits, mushrooms, and seafood, and a low intake of dairy products in both periods. Furthermore, the PCA1 pattern from early to mid-pregnancy was characterized by a low intake of alcoholic beverages. The PCA2 pattern was characterized by a high intake of vegetables, eggs, and dairy products, and a low intake of cereals and meat products in both periods. Further, the PCA2 pattern from early to mid-pregnancy was characterized by a high intake of legumes.

**Table 2:** Factor loadings^a^ and explained variation of dietary patterns identified using RRR, PLS and PCA from pre- to early pregnancy

	RRR	PLS	PCA	
			PCA1	PCA2
Factor loadings of food groups				
Cereals	**0.38**	**0.41**	−0.14	**−0.36**
Pulses	0.02	0.01	**0.33**	0.19
Nuts and seeds	0.14	0.13	0.15	−0.08
Vegetables	−0.04	0.04	**0.48**	**0.22**
Fruits	**0.57**	**0.51**	**0.32**	0.11
Mushroom	0.02	0.07	**0.50**	0.05
Fish and shellfish	0.02	0.05	**0.34**	−0.08
Meat	0.10	0.04	−0.002	**− 0.36**
Eggs	0.11	0.08	−0.11	**0.40**
Milk and dairy products	−0.06	−0.07	**− 0.26**	**0.64**
Confectioneries	−0.14	−0.10	0.12	−0.19
Alcohol beverage	**−0.46**	**−0.58**	− 0.19	−0.04
Non-Alcohol beverage	**−0.50**	**−0.44**	0.15	−0.11
Explained variation (%) in food groups	7.7	8.5	12.7	10.9
Explained variation (%) in birth weight SD score	0.31	0.29	0.0044	0.0001

*RRR* reduced-rank regression, *PLS* partial least-squares regression, *PCA* principal component analysis, *SD* standard deviation

^a^Factor loadings ≧ |0.20| are shown in bold

The explained variation in the predictive variables (food groups) was lower for RRR than PCA, and PLS was in between (from pre- to early pregnancy, RRR: 7.7%, PLS: 8.5%, PCA1: 12.7%, PCA2: 10.9%; from early to mid-pregnancy, RRR: 7.3%, PLS: 8.2%, PCA1: 13.5%, PCA2: 11.6%). The explained variation in the response variable (birth weight SD score) was higher for RRR than PCA, and PLS was in between (from pre- to early pregnancy, RRR: 0.31%, PLS: 0.29%, PCA1: 0.0044%, PCA2: 0.0001%; from early to mid-pregnancy, RRR: 0.50%, PLS: 0.46%, PCA1: 0.0014%, PCA2: 0.014%).

### Association of the dietary patterns with birth weight SD score and SGA

The associations of each dietary pattern with birth weight SD score and SGA from pre- to early pregnancy and from early to mid-pregnancy are shown in Table 3 and Supplementary Table 2, respectively. In both periods, higher adoption of the RRR pattern was significantly associated with higher birth weight SD scores and lower risk of SGA in adjusted model 2 (birth weight SD score: β [Quartile 4 vs. Quartile 1] 0.10 [95%Confidence interval [CI] 0.06, 0.14], *P* for trend < 0.001 from pre- to early pregnancy, β [Quartile 4 vs. Quartile 1] 0.12 [95%CI 0.08, 0.16], *P* for trend < 0.001 from early to mid-pregnancy; risk of SGA: odds ratio [OR] [Quartile 4 vs. Quartile 1] 0.83 [95%CI 0.69, 0.99], *P* for trend = 0.02 from pre- to early pregnancy, OR [Quartile 4 vs. Quartile 1] 0.85 [95%CI 0.71, 1.02], *P* for trend = 0.04 from early to mid-pregnancy).

**Table 3:** Associations of dietary patterns with birth weight SD score and SGA from pre- to early pregnancy

	Quartile 1	Quartile 2		Quartile 3		Quartile 4		*P* for trend
		*β* or OR	95% CI	*β* or OR	95% CI	*β* or OR	95% CI	
**Birth weight SD score,** ***β*** **and 95%CI**								
RRR								
Unadjusted model	Reference	**0.06**	**0.02, 0.10**	**0.09**	**0.05, 0.13**	**0.14**	**0.10, 0.18**	**< 0.001**
Adjusted model 1^a^	Reference	**0.06**	**0.02, 0.10**	**0.09**	**0.05, 0.13**	**0.12**	**0.08, 0.17**	**< 0.001**
Adjusted model 2^b^	Reference	**0.04**	**0.01, 0.08**	**0.08**	**0.04, 0.12**	**0.10**	**0.06, 0.14**	**< 0.001**
PLS								
Unadjusted model	Reference	**0.06**	**0.02, 0.10**	**0.12**	**0.08, 0.16**	**0.13**	**0.09, 0.17**	**< 0.001**
Adjusted model 1	Reference	**0.06**	**0.02, 0.10**	**0.11**	**0.07, 0.15**	**0.12**	**0.08, 0.16**	**< 0.001**
Adjusted model 2	Reference	**0.05**	**0.01, 0.09**	**0.10**	**0.06, 0.13**	**0.09**	**0.05, 0.13**	**< 0.001**
PCA1								
Unadjusted model	Reference	0.04	−0.004, 0.08	0.02	−0.02, 0.06	0.03	−0.01, 0.07	0.33
Adjusted model 1	Reference	0.03	−0.01, 0.07	0.002	−0.04, 0.04	0.01	−0.03, 0.05	0.91
Adjusted model 2	Reference	0.04	−0.003, 0.07	0.01	−0.03, 0.05	0.02	−0.02, 0.06	0.66
PCA2								
Unadjusted model	Reference	−0.03	− 0.07, 0.01	0.002	− 0.04, 0.04	−0.02	− 0.06, 0.02	0.7
Adjusted model 1	Reference	−0.03	−0.07, 0.01	0.001	−0.04, 0.04	− 0.02	−0.06, 0.02	0.65
Adjusted model 2	Reference	−0.02	−0.06, 0.02	0.01	−0.03, 0.05	− 0.003	−0.04, 0.04	0.72
**SGA, OR and 95%CI**								
RRR								
Unadjusted model	Reference	0.86	0.72, 1.01	**0.78**	**0.66, 0.93**	**0.77**	**0.65, 0.91**	**0.001**
Adjusted model 1	Reference	0.87	0.73, 1.03	**0.80**	**0.67, 0.95**	**0.79**	**0.67, 0.95**	**0.006**
Adjusted model 2	Reference	0.88	0.75, 1.05	**0.81**	**0.68, 0.96**	**0.83**	**0.69, 0.99**	**0.02**
PLS								
Unadjusted model	Reference	**0.81**	**0.68, 0.95**	**0.76**	**0.64, 0.90**	**0.73**	**0.62, 0.87**	**< 0.001**
Adjusted model 1	Reference	**0.81**	**0.69, 0.96**	**0.77**	**0.65, 0.91**	**0.74**	**0.62, 0.89**	**< 0.001**
Adjusted model 2	Reference	**0.82**	**0.69, 0.98**	**0.78**	**0.66, 0.93**	**0.77**	**0.64, 0.92**	**0.003**
PCA1								
Unadjusted model	Reference	0.88	0.74, 1.05	0.94	0.80, 1.12	1.01	0.86, 1.20	0.68
Adjusted model 1	Reference	0.89	0.75, 1.06	0.97	0.82, 1.15	1.05	0.89, 1.25	0.40
Adjusted model 2	Reference	0.88	0.74, 1.05	0.95	0.80, 1.13	1.02	0.86, 1.21	0.63
PCA2								
Unadjusted model	Reference	1.04	0.88, 1.23	**0.81**	**0.68, 0.97**	0.97	0.82, 1.15	0.22
Adjusted model 1	Reference	1.05	0.89, 1.24	**0.82**	**0.69, 0.98**	0.98	0.83, 1.17	0.30
Adjusted model 2	Reference	1.04	0.88, 1.23	**0.79**	**0.66, 0.95**	0.95	0.80, 1.13	0.15

*RRR* reduced-rank regression, *PLS* partial least-squares regression, *PCA* principal component analysis, *β* regression coefficients, *OR* odds ratio, *CI* confidence interval, *SGA* small for gestational age

^a^Model 1 adjusted for maternal age (< 25; 25–29; 30–34; ≧ 35, years), pre-pregnancy body mass index (continuous kg/m^2^), educational attainment (high school graduate or less; junior college or vocational college graduate; university graduate or above), household income (< 4,000,000; 4,000,000–5,999,999; ≧ 6,000,000 Japanese Yen/year), cigarette smoking (never; stopped before pregnancy; stopped after pregnancy; current), alcohol drinking (never; former; current), folic acid supplement consumption during early pregnancy (yes; no)

^b^Model 2 adjusted for variables in model 1 plus maternal gestational weight gain (continuous, kg)

Higher adoption of the PLS pattern in both periods was significantly associated with higher birth weight SD scores and lower risk of SGA in adjusted model 2 (birth weight SD score: β [Quartile 4 vs. Quartile 1] 0.09 [95%CI 0.05, 0.13], *P* for trend < 0.001 from pre- to early pregnancy, β [Quartile 4 vs. Quartile 1] 0.14 [95%CI 0.10, 0.18], *P* for trend < 0.001 from early to mid-pregnancy; risk of SGA: OR [Quartile 4 vs. Quartile 1] 0.77 [95%CI 0.64, 0.92], *P* for trend = 0.003 from pre- to early pregnancy, OR [Quartile 4 vs. Quartile 1] 0.76 [95%CI 0.64, 0.91], *P* for trend = 0.001 from early to mid-pregnancy).

The PCA1 pattern was not significantly associated with birth weight SD score or SGA risk in either period. The PCA2 pattern from pre- to early pregnancy was not associated with birth weight SD scores and SGA risk. Although higher adoption to the PCA2 pattern from early to mid-pregnancy was significantly associated with higher birth weight SD scores in adjusted model 2 (β [Quartile 4 vs Quartile 1] 0.05 [95% CI 0.01, 0.09], *P* for trend = 0.01), the pattern was not associated with the risk of SGA.

### Nutrient intakes of participants according to adoption to the dietary patterns

Energy-adjusted nutrient intake stratified by the factor score of each dietary pattern is shown in Supplementary Tables 3, 4, 5 and 6. Each dietary pattern was associated with the intake of a variety of nutrients, and the five nutrients with the largest absolute differences between the fourth and first quartile groups were as follows. Higher adoption to the RRR pattern was associated with a higher intake of beta-cryptoxanthin, carbohydrates, vitamin C, vitamin B1, and copper from pre- to early pregnancy, and with a higher intake of carbohydrates, beta-cryptoxanthin, vitamin C, copper, and dietary fiber from early to mid-pregnancy.

Higher adoption to the PLS pattern was associated with a higher intake of carbohydrates, beta-cryptoxanthin, vitamin C, copper, and vitamin B1 from pre- to early pregnancy, and with a higher intake of carbohydrates, vitamin C, copper, dietary fiber, and beta-cryptoxanthin in early to mid-pregnancy.

Higher adoption to the PCA1 pattern was associated with a higher intake of dietary fiber, iron, vitamin C, folate, and potassium from pre- to early pregnancy, and with a higher intake of dietary fiber, iron, vitamin B6, folate, and potassium from early to mid-pregnancy. Higher adoption to the PCA2 pattern was associated with a higher intake of calcium, phosphorus, vitamin B2, potassium, and pantothenic acid from pre- to early pregnancy, and with a higher intake of potassium, calcium, magnesium, phosphorus, and vitamin B2 from early to mid-pregnancy.

### Sensitivity analysis

When RRR, PLS, and PCA were performed including the excluded subjects with available data on dietary intake and SGA occurrence, milk and dairy products were no longer an important component in the RRR and PLS patterns, while vegetables came to be an important component in the PLS pattern from early to mid-pregnancy. However, the other food groups that characterized the dietary patterns were the same as those in the main analysis (Supplementary Table 7). In addition, the association of each dietary pattern with birth weight SD score and SGA risk were similar as in the main analysis (Supplementary Table 8). Therefore, our main analysis might underestimate the association of vegetable intake with SGA risk and overestimate the association of milk and dairy products intake with SGA risk from early to mid-pregnancy. The excluded subjects had shorter gestational weeks and higher occurrence of hypertensive disorders of pregnancy than those analyzed (Supplementary Table 9), and vegetables and milk and dairy products might have an association with healthy fetal growth in those specific populations.

We compared the characteristics between the included and excluded subjects (Supplementary Table 9) and found that the included subjects were older and had a higher annual household income and educational qualification compared with the excluded subjects. When RRR, PLS, and PCA were performed in the included subjects stratified by maternal age, annual household income, or educational qualification, food groups (such as cereals, fruits, alcoholic beverages, and non-alcoholic beverages) were consistently important components in the RRR and PLS patterns in both periods in almost all subgroups (data not shown). On the other hand, the importance of the other food groups in the RRR and PLS patterns differed in subgroups. Therefore, we believe that even if the excluded subjects are added in the main analysis, the RRR and PLS patterns in both periods would be characterized by a high intake of cereals and fruits and low intake of alcoholic and non-alcoholic beverages.

In addition, in the association of the RRR and PLS patterns with birth weight SD score and SGA risk, the estimated β and OR in all subgroups, except those with an educational qualification of university graduate or above, were comparable to those in the main analysis (some of these associations were not statistically significant, but this was considered to be due to the smaller number of subjects in the subgroup analyses compared to the main analysis) (data not shown). In subjects with an educational qualification of university graduate or above, the association of the RRR and PLS patterns in both periods with birth weight SD score and SGA risk was stronger than that in the main analysis. These individuals had lower pre-pregnancy BMI and weight gain during pregnancy than those in the main analysis (data not shown). Undereating owing to the desire to stay slim among Japanese women is a problem leading to a downward trend in the birth weights of infants [5]. It might be possible that those with an educational qualification of university graduate or above were more likely to have such an orientation and appropriate dietary patterns had significant impacts on their SGA risk.

We also performed RRR, PLS, and PCA in subjects without GDM and found that the food groups that characterized the dietary patterns were almost the same as those in the main analysis (differences in factor loadings were less than 0.1 in absolute values, Supplementary Table 10). Further, the associations of dietary patterns with birth weight SD score and SGA risk were almost the same as those in the main analysis (Supplementary Table 11). We believe that the confounding effect of GDM was small.

## Discussion

### Main finding

This is the first report to compare the association of dietary patterns in pregnant women derived from RRR, PLS, and PCA with birth weight SD score and SGA risk of their children. The dietary patterns extracted by RRR and PLS were similar and characterized by a high intake of cereals and fruits and a low intake of alcoholic and non-alcoholic beverages in both pre- to early pregnancy and early to mid-pregnancy. Furthermore, the RRR pattern from early to mid-pregnancy was characterized by a high intake of dairy products, while the PLS pattern in both periods was characterized by a high intake of dairy products and mushrooms. Higher adoption to both the RRR and PLS patterns were associated with an increased birth weight SD score and lower risk of SGA. In contrast, the PCA1 pattern was not associated with birth weight SD score or SGA risk in either period. Although the PCA2 pattern was associated with increased birth weight SD scores from early to mid-pregnancy, no other associations with birth weight SD score or SGA risk were observed.

### Discussion on dietary patterns

To our knowledge, there have been no previous studies on the association between dietary patterns and characteristics, such as the RRR and PLS patterns, and birth weight-related outcomes. Regarding individual food groups, there are several reports on the association between each food group and birth weight-related outcomes. In our previous study, we reported that a higher intake of fruits and cereals from pre- to early pregnancy and early to mid-pregnancy was associated with higher birth weight and lower risk of low birth weight (LBW) [28, 29]. Regarding dairy products, Heppe et al. found in a prospective study in the Netherlands that the intake of milk during early pregnancy (FFQ was completed at around 13.5 weeks of gestation; it referred to the previous 3 months) found no association with SGA risk [30]. On the other hand, Olsen et al. reported in a prospective cohort study in Denmark that a higher intake of milk during mid-pregnancy (FFQ was completed at 25 weeks of gestation; it referred to the previous 4 weeks) was associated with a lower risk of SGA [31]. A meta-analysis by Patra et al. reported that consumption of high doses of pure alcohol (10 g/day) during pregnancy monotonically increased the risk of SGA [32]. The components of beverages in our study included caffeine-rich beverages such as coffee and tea and sugar-rich beverages such as carbonated beverages. A meta-analysis by Greenwood et al. reported that every 100 mg/day increase in caffeine intake during pregnancy increased the risk of SGA by 10% [33]. A prospective cohort study in Norway by Grundt et al. reported that increased consumption of sugar-sweetened carbonated beverages during pregnancy was associated with decreased birth weight and increased risk of LBW among women without gestational diabetes [34]. In light of the above, the individual food groups comprising the dietary patterns extracted by RRR and PLS in our study are reasonable, and each of these food groups is considered to be associated with birth weight SD score and SGA, even after accounting for the effects of other food groups. In addition, since multiple food groups associated with diseases can be identified as dietary patterns, our results may be useful in suggesting dietary habits to prevent SGA.

In several previous studies on dietary patterns derived from PCA, the “Healthy Japanese pattern” with high intake of vegetables, fruits, legumes, mushrooms, seaweed, and seafood was extracted as a typical dietary pattern of the Japanese people and was associated with a lower risk of hypertension, diabetes, cardiovascular disease, physical dysfunction, and mortality [35]. Epidemiological studies by Knudsen et al. and Thompson et al. reported that the ‘Health-conscious pattern: characterized by high intake of vegetables, fruits, fish, and poultry and the ‘Traditional pattern: characterized by high intake of vegetables, fruits, legumes, and dairy products were associated with lower risk of SGA, respectively [15, 16]. Therefore, it was expected that the PCA1 pattern in the present study, which was characterized by a high intake of vegetables, fruits, legumes, mushrooms, and seafood, and low intake of dairy products, would also be associated with birth weight SD score and SGA. However, the PCA1 pattern was not associated with birth weight SD score or SGA. This might be because the PCA1 pattern had quite different characteristics from the RRR pattern; that is, the PCA1 pattern did not contain enough characteristics of food groups contained in the RRR pattern (such as cereals, dairy products, and non-alcoholic beverages). Additionally, the PCA1 pattern contained many characteristics of food groups that the RRR pattern did not contain (such as legumes, vegetables, and seafood).

The PCA2 pattern was characterized by a high intake of dairy products, eggs, vegetables, and legumes (only from early to mid-pregnancy) and a low intake of cereals and meat. This dietary pattern was similar to the “Western dietary pattern: characterized by high intake of dairy products, eggs, vegetables, fruits, and cereals” in a previous study among Japanese pregnant women [36]. Thus, the PCA2 pattern was considered as a common dietary pattern among them. The PCA2 pattern in the present study also had different characteristics from the RRR pattern. Both dietary patterns were characterized by a high intake of dairy products in early to mid-pregnancy, while other factors were not common. As a result, we believe that the PCA2 pattern also showed little association with birth weight SD score and SGA.

In general, the dietary pattern extracted by PCA reflects people’s actual dietary conditions, while it may have little association with the outcomes. However, although the dietary patterns extracted by RRR or PLS do not necessarily reflect people’s actual dietary conditions, they are more likely to be associated with the outcomes. Therefore, although the actual dietary patterns of the pregnant women in this study were not associated with SGA, the dietary patterns revealed by RRR and PLS may be useful as a benchmark for future ideal dietary patterns to prevent SGA.

### Discussion on nutrient intake

Regarding nutrient intake, the dietary patterns extracted by RRR and PLS were characterized by a high intake of carbohydrates, vitamin B1, vitamin C, beta-cryptoxanthin, and copper (Supplementary Tables 3, 4, 5 and 6). Low maternal blood glucose concentration during pregnancy has been reported to decrease birth weight and increase the risk of LBW [37]. Glucose is the primary source of energy for fetal growth and the fetus obtains most of the glucose from the mother by placental transport, since the fetus has little glycogenesis [38, 39]. The placental transport of glucose is proportional to maternal blood glucose concentration and placental erythrocyte flow rate, and the increase in maternal blood glucose concentration is due to maternal hepatic gluconeogenesis and diet (carbohydrates being the main source). Therefore, this study suggests that it is important to consume sufficient amounts of carbohydrates during pregnancy to decrease SGA risk. However, the association between carbohydrate intake and GDM risk should be noted. Looman et al. examined the association of pre-pregnancy intake of carbohydrate-rich food groups and GDM risk and reported that higher cereal intake was associated with a higher risk of GDM, whereas fruits and fruit juice intakes were inversely associated [40]. Therefore, the source of carbohydrates should also be considered.

Thiamine, also known as vitamin B1, is a water-soluble vitamin and an important cofactor in several biochemical pathways involved in glucose metabolism [41]. The requirement for thiamine is increased in pregnant mothers, and it has been reported that about 50% of pregnant women develop biochemical thiamine deficiency [42]. It has been suggested that thiamine deficiency causes intrauterine fetal growth retardation. For example, a study by Heinze et al. in Germany reported that pregnant women with complications of intrauterine fetal growth retardation had significantly lower erythrocyte thiamine concentrations than normal pregnant women [43]. Furthermore, chronic alcohol intake causes a decrease in thiamine absorption in the gastrointestinal tract [44]. Tannin, a polyphenol present in coffee and tea, has been reported to inactivate thiamine [45, 46]. Therefore, dietary patterns such as high intake of thiamine and low intake of alcohol and preferred beverages (such as coffee and tea) during pregnancy may be important in preventing SGA from the perspective of efficient glucose (carbohydrate) utilization.

Oxidative stress is defined as an imbalance between oxidants and antioxidants, with the predominance of oxidants, and has adverse effects on the course and outcome of pregnancy, including eclampsia, fetal growth retardation, and preterm birth, which result in SGA [47]. Vitamin C exerts antioxidant effects by scavenging reactive oxygen and nitrogen species and plays an important role in protection against oxidative stress, which increases during pregnancy [48, 49]. Saker et al. reported that newborns with SGA and their mothers had lower concentrations of antioxidant activity (oxygen radical absorbance capacity, ORAC), vitamin C, and vitamin E in their blood, and higher concentrations of oxidative stress markers (hydroperoxides and carbonyl proteins) compared to newborns with appropriate for gestational age and their mothers [50]. Unlike many other animal species, humans are unable to biosynthesize ascorbic acid [51]; thus, it is important to obtain vitamin C from the diet. Beta-cryptoxanthin is one of the six major carotenoids (lutein, zeaxanthin, alpha-carotene, beta-carotene, lycopene, and beta-cryptoxanthin) measured in human serum, which is mainly consumed in oranges, orange juice, and tangerines [52]. Beta-cryptoxanthin, like other carotenoids, is an antioxidant that has been reported to prevent free radical damage to biomolecules such as nucleic acids, lipids, and proteins [53]. Although there are few reports investigating the association between beta-cryptoxanthin and SGA risk, Cohen et al. reported in a nested case-control study in Canada that maternal blood beta-cryptoxanthin concentration at 24–26 weeks of gestation was associated with a lower risk of SGA, and the present study supported this association [54]. However, there is insufficient evidence on the association of beta-cryptoxanthin with SGA, and further studies are needed. Although the intake of vitamin C and beta-cryptoxanthin may be important in the prevention of SGA from the viewpoint of inhibiting oxidative stress, it should be noted that these nutrients are abundant in fruits and that the present study did not include vegetables rich in these nutrients in the RRR or PLS patterns. That is, the present results may reflect the association of other components in fruits with SGA (such as carbohydrates and micronutrients).

### Limitations and strengths

This study had some limitations. First, the included subjects were older and had a higher annual household income and educational qualification compared with the excluded subjects, resulting in a possible selection bias. Since the association of the RRR and PLS patterns with birth weight SD score and SGA risk in subjects with an educational qualification of university graduate or above in the subgroup analysis was stronger than in the main analysis, our main analysis might overestimate the association. Second, measurement errors in food and nutrient intake were inevitable, since the FFQ was used to assess dietary intake. Third, the FFQ used in this study was a partially modified version of the FFQ used in the JPHC Study, which has been validated in a Japanese population, with the addition of the response option “constitutionally unable to eat” to the question regarding food intake frequency. However, since this option was treated as equivalent to “less than once a month,” it was considered to have little effect on the interpretation of the results.

The strengths of this study are as follows: First, the TMM BirThree Cohort study is a prospective birth cohort study with a large sample size, which enabled us to assess the association between dietary patterns and birth weight-related outcomes. Second, by using FFQs from two different periods, we were able to identify dietary patterns that were associated with birth-related outcomes before and during pregnancy. Third, by using the RRR, PLS, and PCA methods, we were able to identify dietary patterns that were associated with birth weight-related outcomes, which differed significantly from people’s actual dietary patterns.

## Conclusions

The dietary patterns with high intakes of cereals and fruits and low intakes of alcoholic and non-alcoholic beverages before and during pregnancy extracted by RRR and PLS were associated with a decreased SGA risk in Japan, while dietary patterns extracted by PCA were not associated with SGA risk. Although the actual dietary patterns of the pregnant women in this study were not associated with SGA, the dietary patterns revealed by RRR and PLS may be useful as a benchmark for future ideal dietary patterns to prevent SGA.

## Supplementary Information

**Additional file 1: Supplementary Table 1.** Factor loadings and explained variation of dietary patterns identified using RRR, PLS and PCA from early to mid pregnancy. **Supplementary Table 2.** Associations of dietary patterns with birth weight SD score and SGA from early to mid pregnancy. **Supplementary Table 3.** Characteristics of participants stratified by the adoption to the RRR patterns. **Supplementary Table 4.** Characteristics of participants stratified by the adoption to the PLS patterns. **Supplementary Table 5.** Characteristics of participants stratified by the adoption to the PCA1 patterns. **Supplementary Table 6.** Characteristics of participants stratified by the adoption to the PCA2 patterns. **Supplementary Table 7.** Factor loadings and explained variation of dietary patterns identified using RRR, PLS and PCA with the excluded subjects who had available data on dietary intake and incidence of SGA (*n* = 18,667). **Supplementary Table 8.** Associations of dietary patterns with birth weight SD score and SGA with the excluded subjects who had available data on dietary intake and incidence of SGA (*n* = 18,667). **Supplementary Table 9.** The characteristics of the 17,728 mothers who were analyzed and the 4765 mothers who were excluded from the analysis. **Supplementary Table 10.** Factor loadings and explained variation of dietary patterns identified using RRR, PLS and PCA in subjects without gestational diabetes mellitus (*n* = 17,325). **Supplementary Table 11.** Associations of dietary patterns with birth weight SD score and SGA in subjects without gestational diabetes mellitus (*n* = 17,325).

**Additional file 2.**

## Data Availability

The datasets analyzed in this study are available from the corresponding author upon reasonable request.

## Abbreviations

BMI: Body mass index
CI: Confidence interval
FFQ: Food frequency questionnaire
JPHC Study: Japan Public Health Center-based Prospective Study
LBW: Low birth weight
OR: Odds ratio
PCA: Principal component analysis
PLS: Partial least squares
PRESS: Predicted residual sum of squares
RRR: Reduced rank regression
SD: Standard deviation
SGA: Small for gestational age
TMM BirThree Cohort Study: Tohoku Medical Megabank Project Birth and Three-Generation Cohort Study
ToMMo: Tohoku Medical Megabank Organization
