# Supplementary material for: Dietary patterns before and during pregnancy and small for gestational age in Japan: a prospective birth cohort study
Source: Nutr J. 2022 Sep 16;21:57. doi: 10.1186/s12937-022-00808-7 (PMC9479276; doi:10.1186/s12937-022-00808-7)
Supplement: Supplementary file 2 — Additional file 2. [file 12937_2022_808_MOESM2_ESM.docx]

**Supporting information 1. Details on dietary information**

To investigate dietary intake before and during pregnancy, we used two semi-quantitative food frequency questionnaires (FFQs). The first FFQ was administered during early pregnancy to evaluate dietary habits from pre-pregnancy to early pregnancy which asked about dietary habits in the past 12 months. The second FFQ was administered during mid-pregnancy to evaluate dietary habits from the early to mid-pregnancy period which asked about dietary habits since the first FFQ. The mean response periods of the first and second FFQs were 20.6 ± 7.8 weeks and 28.5 ± 5.8 weeks of gestation, respectively. These FFQs were used to estimate the mothers’ usual intake frequency and portion sizes of 130 food items and are based on the FFQ from the Japan Public Health Center-based Prospective Study (JPHC Study) [1, 2]. The response option ‘constitutionally unable to eat it’ for individual food items is unique to the FFQ of our study, which covers the case where a respondent is unable to eat food items due to allergies or other symptoms, rather than simply not eating them.

The frequency of intake options consisted of: “constitutionally unable to eat it,” “never or less than once a month,” “from one to three times a month,” “once or twice a week,” “three or four times a week,” “five or six times a week,” “once a day,” “two or three times a day,” “from four to six times a day,” and “seven or more times a day.” When values of the frequency of intake were missing, they were complemented with “never or less than once a month.” The standard amounts per serving of each food item were presented in pictures or texts, and the portion sizes were specified as multiplies of the standard amount (less than half, one, and more than one and a half times). When values of portion size were missing, they were complemented with “one.” The daily intake of each food and beverage item was calculated by multiplying the frequency by the portion size. Based on the Standard Tables of Food Composition in Japan (Fifth Revised and Enlarged Edition 2005) [3], foods were classified into 18 food groups, and the daily intake of each nutrient was calculated. In this study, we selected 13 food groups and 28 nutrients with Spearman's correlation coefficients of 0.3 or higher between estimated intakes measured by the FFQ and the dietary record method [4-7]. The intake of each food group was adjusted for energy intake using the residual method [8] and then standardized by subtracting the mean value and dividing by the standard deviation of each food group intake.

**Supporting information 2. Details on dietary patterns**

Dietary patterns from pre- to early pregnancy and from early to mid-pregnancy were extracted using RRR, PLS, and PCA, respectively. RRR, PLS, and PCA all utilized PROC PLS in SAS (version 9.4, SAS Institute Inc., Cary, North Carolina), changing the method option to reflect each desired method. A more detailed description of their application in nutritional epidemiology has been reported by Hoffman et al. [9]. RRR, PLS, and PCA derive the patterns of predictor variables that best explain the variation in response variables, predictor variables and response variables, and predictor variables, respectively. In the present study, we set the intake of 13 food groups as predictor variables and the birth weight SD score as a response variable, which was a continuous variable associated with SGA risk. The final number of factors was selected by applying random sample cross-validation, which is an option to select the optimal number of factors included in PROC PLS [10]. The number of factors selected represents the model with the smallest predicted residual sum of squares (PRESS). In this study, the number of factors with the minimum PRESS was two; thus, the number of extracted factors was set to two in PCA. On the other hand, because the number of extracted factors cannot be larger than the rank of the corresponding covariance matrix in RRR [9], the number of extracted factors was set to one, which was the number of response variables in RRR. The number of extracted factors in PLS was also set to one to facilitate a comparison of the results with RRR.

Factor loadings on food groups obtained by RRR, PLS, and PCA represent the degree of contribution of that food group to the dietary pattern [11]. Food groups with absolute factor loadings of 0.2 or higher were considered to characterize the dietary pattern [12]. To calculate factor scores, each subject's food intake was weighted by factor loadings and summed, which indicated the degree of adoption to a dietary pattern [11]. They were divided into quartiles (Quartile 1: lowest adoption; Quartile 4: highest adoption) and used for analysis in association with birth weight SD score and SGA risk.

**Supporting information 3. Details on potential confounders**

Potential confounders for the association between dietary patterns and birth outcomes were determined based on previous reports [13-15]. Maternal age at delivery was obtained from the medical records and used as a categorical variable (< 25, 25–29 (reference category), 30–35, and ≥ 35 years). Pre-pregnancy body mass index (BMI) was calculated by dividing the weight (kg) by the square of the height (m^2^) before pregnancy obtained from the medical record and used as a continuous variable. Maternal weight gain during pregnancy was calculated by subtracting maternal weight before pregnancy from maternal weight before delivery and was used as a continuous variable. Values of maternal height less than 100 cm or more than 250 cm (n=2 [0.01%]), and values of maternal weight before delivery less than 20 kg or more than 200 kg (n=5 [0.03%]) were medically implausible, and we treated them as missing values. Parity was obtained from medical records and used as a binary variable (one or more, and never). Educational qualification was obtained from a questionnaire 12 months after mothers’ delivery and was divided into three categories: high school graduate or less (elementary, junior high school, or senior high school), college graduate (2-year college or special training school), and university graduate or above (university or graduate school). Annual household income was obtained from a questionnaire during mid-pregnancy and used as a categorical variable (< 4,000,000, 4,000,000–5,999,999, and ≥ 6,000,000 Japanese yen per year). Habits of smoking, drinking, and taking folic acid supplements were obtained from a questionnaire during early pregnancy and used as categorical variables (never, quit before pregnancy, quit after noticing pregnancy and current), (never, former, and current), and (yes and no), respectively.

**References**

1. Takachi R, Ishihara J, Iwasaki M, Hosoi S, Ishii Y, Sasazuki S, et al. Validity of a self-administered food frequency questionnaire for middle-aged urban cancer screenees: Comparison with 4-day weighed dietary records. J Epidemiol. 2011;21:447-58. <https://doi.org/10.2188/jea.je20100173>

2. Watanabe S, Tsugane S, Sobue T, Konishi M, Baba S. Study design and organization of the JPHC study. Japan Public Health Center-based Prospective Study on Cancer and Cardiovascular Diseases. J Epidemiol. 2001;11;Suppl:S3-7. <https://doi.org/10.2188/jea.11.6sup_3>

3. Ministry of Education Culture Sports Science and Technology. Standards: Tables of food composition in Japan (fifth revised and enlarged edition) (in Japanese); 2005. <https://www.mext.go.jp/b_menu/shingi/gijyutu/gijyutu3/toushin/05031802.htm>. Accessed 14 Aug 2021

4. Ishihara J, Sobue T, Yamamoto S, Yoshimi I, Sasaki S, Kobayashi M, et al. Validity and reproducibility of a self-administered food frequency questionnaire in the JPHC Study Cohort II: Study design, participant profile and results in comparison with Cohort I. J Epidemiol. 2003;13;Suppl:S134-47. <https://doi.org/10.2188/jea.13.1sup_134>

5. Tsugane S, Kobayashi M, Sasaki S, JPHC. Validity of the self-administered food frequency questionnaire used in the 5-year follow-up survey of the JPHC Study Cohort I: Comparison with dietary records for main nutrients. J Epidemiol. 2003;13;Suppl:S51-6. <https://doi.org/10.2188/jea.13.1sup_51>

6. Sasaki S, Kobayashi M, Tsugane S, JPHC. Validity of a self-administered food frequency questionnaire used in the 5-year follow-up survey of the JPHC Study Cohort I: Comparison with dietary records for food groups. J Epidemiol. 2003;13;Suppl:S57-63. <https://doi.org/10.2188/jea.13.1sup_57>

7. Ishihara J, Inoue M, Kobayashi M, Tanaka S, Yamamoto S, Iso H, et al. Impact of the revision of a nutrient database on the validity of a self-administered food frequency questionnaire (FFQ). J Epidemiol. 2006;16:107-16. <https://doi.org/10.2188/jea.16.107>

8. Willett W, Stampfer MJ. Total energy intake: Implications for epidemiologic analyses. Am J Epidemiol. 1986;124:17-27. <https://doi.org/10.1093/oxfordjournals.aje.a114366>

9. Hoffmann K, Schulze MB, Schienkiewitz A, Nöthlings U, Boeing H. Application of a new statistical method to derive dietary patterns in nutritional epidemiology. Am J Epidemiol. 2004;159:935-44. <https://doi.org/10.1093/aje/kwh134>

10. Naja F, Itani L, Hwalla N, Sibai AM, Kharroubi SA. Identification of dietary patterns associated with elevated blood pressure among Lebanese men: A comparison of principal component analysis with reduced rank regression and partial least square methods. PLOS ONE. 2019;14:e0220942. <https://doi.org/10.1371/journal.pone.0220942>

11. Sauvageot N, Leite S, Alkerwi A, Sisanni L, Zannad F, Saverio S, et al. Association of empirically derived dietary patterns with cardiovascular risk factors: A comparison of PCA and RRR methods. PLOS ONE. 2016;11:e0161298. <https://doi.org/10.1371/journal.pone.0161298>

12. Batis C, Mendez MA, Gordon-Larsen P, Sotres-Alvarez D, Adair L, Popkin B. Using both principal component analysis and reduced rank regression to study dietary patterns and diabetes in Chinese adults. Public Health Nutr. 2016;19:195-203. <https://doi.org/10.1017/S1368980014003103>

13. Chatzi L, Mendez M, Garcia R, Roumeliotaki T, Ibarluzea J, Tardón A, et al. Mediterranean diet adherence during pregnancy and fetal growth: INMA (Spain) and RHEA (Greece) mother–child cohort studies. Br J Nutr. 2012;107:135-45. <https://doi.org/10.1017/S0007114511002625>

14. Lu MS, Chen QZ, He JR, Wei XL, Lu JH, Li SH, et al. Maternal dietary patterns and fetal growth: A large prospective cohort study in China. Nutrients. 2016;8. <https://doi.org/10.3390/nu8050257>

15. Englund-Ögge L, Brantsæter AL, Juodakis J, Haugen M, Meltzer HM, Jacobsson B, et al. Associations between maternal dietary patterns and infant birth weight, small and large for gestational age in the Norwegian Mother and Child Cohort Study. Eur J Clin Nutr. 2019;73:1270-82. <https://doi.org/10.1038/s41430-018-0356-y>
